# The Soluble Heparin-Binding EGF-Like Growth Factor Stimulates EGF Receptor Trafficking to the Nucleus

**DOI:** 10.1371/journal.pone.0127887

**Published:** 2015-05-27

**Authors:** Nataliia V. Korotkevych, Andrii Ju. Labyntsev, Denis V. Kolybo, Serhiy V. Komisarenko

**Affiliations:** Molecular Immunology Department, Palladin Institute of Biochemistry of the National Academy of Sciences of Ukraine, Kyiv, Ukraine; Universidad del Pais Vasco, SPAIN

## Abstract

Most ligands of epidermal growth factor receptor (EGFR) have the ability to induce EGFR translocation into the nucleus, where EGFR acts as an important transcriptional regulator. Soluble form of heparin-binding EGF-like growth factor (sHB-EGF) is one of the ligands for EGFR in many cell types; however, there is no evidence for the ability of sHB-EGF to induce EGFR nuclear importation. Here, we demonstrated that treatment of A431 cells with sHB-EGF resulted in nuclear localization of EGFR and such translocation occurs via retrograde pathway. It was shown by confocal microscopy and co-immunoprecipitation assay that the translocation complex consisted of both ligand and receptor. The chromatin immunoprecipitation assay showed the association of sHB-EGF–EGFR complex with promoter region of cyclin D1 in the cell nucleus and this association was prevented by application of EGFR kinase inhibitor AG-1478. The obtained data suggest that sHB-EGF acts similarly to other EGFR ligands and is capable to induce EGFR nuclear translocation as a part of ligand-receptor complex in a tyrosine phosphorylation-dependent manner.

## Introduction

The heparin-binding EGF-like growth factor (HB-EGF) is a member of the EGF family of growth factors. It has high affinity for heparin and heparan sulfate [1]. HB-EGF precursor is synthesized as type I transmembrane protein (pro-HB-EGF), its cleavage by extracellular proteases results in shedding of soluble form of HB-EGF (sHB-EGF). sHB-EGF mediates paracrine and autocrine activation of the EGFR family receptors ErbB1 (EGFR) and ErbB4 promoting survival, proliferation, and migration of different cell types [2]. These autocrine and paracrine activities of sHB-EGF may serve as tumor-promoting factors. Now, it is clear that the mode of action of soluble HB-EGF and proHB-EGF are not similar and that several biological activities of HB-EGF are restricted to the soluble form (e.g. the ability to induce autocrine activation of EGFR).

The ligand binding to EGFR results in EGFR activation, internalization and further recycling or lysosomal degradation of ligand-receptor complex [3]. However, some of EGFR members such as ErbB1 (EGFR) [4], ErbB2 [5] and ErbB3 [6] have been detected in the nucleus as full-length forms. Nuclear localization of these receptors has also been found in different normal [7–9] or malignant cells [10]. All ErbB proteins contain nuclear localization signal (NLS), which is required for the transportation of the receptors to the nucleus through the nuclear pore complex [11]. In the nucleus, these proteins probably regulate transcription of specific target genes by direct binding to transcription regulators [12]. For example, nuclear EGFR acts as a transcription factor resulting in activation of genes required for highly proliferating activities. Upon EGF stimulation, EGFR translocates to the nucleus and after binding to the proximal region of the cyclin D1 promoter stimulates its expression that results in cell proliferation [10]. Interestingly, EGFR itself lacks a DNA-binding domain, that is why its association with other DNA-binding factors, like STAT3 or STAT5, is required for EGFR ability to activate specific genes [13,14].

The HB-EGF precursor was reported to be localized within tumor cell nuclei in human transitional cell carcinoma [15]. The nuclear proHB-EGF can be exported from the nucleus under the effect of reactive oxygen species (ROS) [16]. However, there is no evidence about nuclear localization of sHB-EGF, as well as the ability of sHB-EGF to induce EGFR nuclear translocation upon binding. We addressed these questions in the present study and also revealed the mechanisms of sHB-EGF/EGFR complex trafficking to the perinuclear region.

## Materials and Methods

### Cell culture

A431 cells [17] were received from the cell lines bank of R.E. Kavetskiy Experimental Pathology, Oncology and Radiobiology Institute of NAS of Ukraine. Cells were cultured in high-glucose RPMI-1640 with L-glutamine, supplemented with 10% fetal bovine serum (FBS) and penicillin-streptomycin-amphotericin B (Sigma-Aldrich, Missouri, USA). For microscopy experiments, cells were plated overnight onto cover glasses placed in 35-mm dishes.

### Plasmid constructs

Construction of the plasmids encoding recombinant soluble form of human HB-EGF (sHB-EGF) and mCherry-sHB-EGF was described earlier [18,19]. Briefly, both constructions were based on pET28a plasmid and *E*.*coli* expression strain BL21 DE3 Rosetta (Clonetech, USA). Complementary DNA encoding the full-length form of HB-EGF from U937 cells was used for amplification of fragment encoding a soluble form of sHB-EGF. Amplified gene fragment was merged with expression vector *pET28a* using BamHI and XhoI restriction sites. Therefore, gene construction *pET28a-sHB-EGF* has been obtained. To obtain *pET-28a-mCherry-sHB-EGF*, mCherry encoding sequence was amplified from plasmid *pmCherry* (Clonetech, USA) and inserted into *pET28a-sHB-EGF* at the 5’-terminus of sHB-EGF using XhoI and PstI restriction sites. All molecular cloning reagents were purchased from Thermo Scientific (MA, USA).

*pEGFR-EYFP* plasmid was kindly provided by Prof. V. Verkhusha (Albert Einstein College, NY, USA) and *p23-EYFP* plasmid was kindly provided by Prof. J. Rędowicz (Nencki Institute of Biochemistry, Warsaw, Poland). Transfection of A431 cells with *pEGFR-EYFP* or *p23-EYFP* plasmids were performed with LipofectAMINE LTX (Invitrogen, CA, USA).

### Recombinant protein production

For production of recombinant sHB-EGF and mCherry-sHB-EGF we used *E*.*coli* expression strain BL21 DE3 Rosetta under standard cultivation protocols. Over-night cultures diluted 1:20 with LB media containing 2% glucose (Sigma-Aldrich, Missouri, USA) were cultivated to achieve a cell-density of OD_600_ = 0,5–0,7 at 37°C. After IPTG-induction, the bacterial cells were cultured at the optimized temperature for a 3–3.5 hours before harvesting. The purification of recombinant proteins from bacteria was performed by scientific and production enterprise “Enamine” (Kyiv, Ukraine) on FPLC with Ni-NTI agarose (Qiagen, Limburg, Netherlands) according to the manufacturer recommendations. Purity of recombinant proteins was verified with SDS-PAGE under denaturation conditions.

### Mytogenic activity of sHB-EGF and mCherry-sHB-EGF

Bioactivity of recombinant proteins sHB-EGF and mCherry-sHB-EGF was previously shown [18,19]. In brief, mytogenic activity of human sHB-EGF and mCherry-sHB-EGF was evaluated with MTT proliferation assay on mouse fibroblasts BALB/c 3T3 (Cell lines bank of R.E. Kavetskiy Experimental Pathology, Oncology and Radiobiology Institute of NAS of Ukraine). Both recombinant proteins were able to promote growth and migration of 3T3 cells up to 30–35% compared to untreated cells. The specificity of sHB-EGF effect was proved with EGFR kinase activity inhibitor AG1478 (Sigma-Aldrich, Missouri, USA) and matrix metalloproteinases (MMPs) inhibitor GM6001 (Sigma-Aldrich, Missouri, USA). GM6001 was used to inhibit autocrine sHB-EGF production by cultured cells. It was shown that mCherry-sHB-EGF was able to induce phosphorylation of Erk 1/2 (#4370, Cell signaling technology, USA) in 3T3 cells and p38 MAPK (sc-7149, Santa Cruz Biotech, USA) in A431 cells. Ability of mCherry-sHB-EGF to bind specifically to the receptor on surface of A431 cells was confirmed by confocal microscopy with anti-EGFR (E2760, Sigma-Aldrich, Missouri, USA) and secondary FITC-conjugated antibody (F5387, Sigma-Aldrich, Missouri, USA).

### Cellular fractionation and Western blotting analyses of cell fractions

The antibodies used in this study were as follows: anti-Lamin B1 (#33–2000, Invitrogen, USA), anti-EGFR mAb (E2760, Sigma-Aldrich, Missouri, USA), anti-beta-actin mAb (8226, Abcam, USA), anti-GST (A7340, Sigma-Aldrich, Missouri, USA), total Erk 1/2 (sc-135900, Santa Cruz Biotech, USA), total p38 (sc-7149, Santa Cruz Biotech, USA) Secondary peroxidase conjugated antibodies anti-rabbit IgG (A0545) and anti-mouse IgG (A9044) were obtained from Sigma-Aldrich (Missouri, USA).

A431 cells were serum-starved and after 24 hours were stimulated with sHB-EGF (500 ng ml-1) for various periods of time. In the case of application of AG1478, the cells were pretreated with this agent during 5 min before sHB-EGF was added. Stimulated cells were disrupted by addition of a cell membrane lysis buffer (10 mM HEPES, 1,5 mM MgCl_2_, 10 mM KCl, 0,5 M DTT, 0,05% NP40 (all from Sigma-Aldrich, Missouri, USA), pH 7.9, Halt Protease Inhibitor Cocktail (Thermo Scientific, MA, USA) and incubated on ice for 10 min. Cells were then passed 20 times through a 25-gaugee needle and centrifuged at 1,500 g for 5 min to sediment the nuclei. The supernatant was again centrifuged at maximum speed of 13,000 g for 20 min, and the resulting supernatant formed the non-nuclear fraction. The nuclear pellet was washed three times with a cell membrane lysis buffer to remove any contamination from cytoplasmic membranes. To exclude the possibility of contamination of nuclear extracts with non-nuclear EGFR, the nuclei from non-stimulated and sHB-EGF-stimulated A431 cells were mixed with non-nuclear fraction of non-stimulated and sHB-EGF-stimulated A431 cells respectively. After that, the nuclei were again centrifuged at maximum speed and washed three times with a cell membrane lysis buffer. The isolated nuclei were resuspended in nuclear lysis buffer (5 mM HEPES, 1,5 mM MgCl2, 0,2 mM EDTA, 0,5 mM DTT, 26% glycerol, 300 mM NaCl) and the mixture was sonicated briefly to aid nuclear lysis and extract nuclear proteins. Nuclear lysates were then collected after centrifugation at 13,000 g for 20 min. Protein concentration was determined with Bradford method [20], and then the samples were mixed with Laemmli sample buffer [21] and heated at 950C for 15 min. Samples were subjected to SDS-PAGE in 8% polyacrylamide gels with subsequent transfer to nitrocellulose membranes. Membranes were probed with monoclonal or polyclonal antibodies followed by horseradish peroxidase-labeled secondary antibodies. Immunoreactive protein bands were detected with an enhanced chemiluminescence reagent (Sigma-Aldrich, Missouri, USA).

### Indirect immunofluorescence and confocal microscopy

Cells seeded on cover glasses at low density were treated with sHB-EGF (1.5 ug/ml) or mCherry-sHB-EGF (5 ug/ml) in 1% BSA/PBS solution during different time periods and fixed with 4% paraformaldehyde (Sigma-Aldrich, Missouri, USA). The optimal concentration of recombinant sHB-EGF was determined by MTT-test. The optimal concentration of mCherry-sHB-EGF for confocal microscopy staining was determined by flow cytometry analysis of A431 cells. In case of application of AG1478 inhibitor, the cells were pretreated during 5 min before sHB-EGF or mCherry-sHb-EGF was added. Cell nucleus was stained with Hoechst 33342 (Sigma-Aldrich, Missouri, USA). Glasses with cells were mounted in DABCO/PVA mounting medium (Sigma-Aldrich, Missouri, USA) and analyzed with LSM 510 META laser scanning confocal microscope (Carl Zeiss, Oberkochen, Germany). Image analysis was done with FIJI software [22]. Images was equally to whole panel normalized with level tool. Next, scale bar and z-stack depth was added to images. ROIs (regions of interest) was cropped if needed and their position was marked. Next, channels was splited into separate images and colocalization was calculated with RG2B plugin.

### Chromatin immunoprecipitation assay (ChIP)

Rabbit serum was obtained from intact rabbit *Oryctolagus cuniculus* Soviet chinchilla in strict accordance with the recommendations of the Guide for the Care and Use of Laboratory Animals of the National Institutes of Health. The protocol was approved by the Committee on the Ethics of Animal Experiments of the Palladin Institute of Biochemistry of NAS of Ukraine (Protocol #9 from 25.10.2011).

A431 cells plated in five 10 cm dishes were serum-starved for 24h and stimulated with sHB-EGF (500 ng ml-1), GST-sHB-EGF (1000 ng ml-1) or 0,01% H2O2 for 1 h. In case of application of AG1478 or PAO (phenylarsine oxide) (Sigma-Aldrich, Missouri, USA) cells were pretreated with inhibitors during 5 min before addition of sHB-EGF or GST-sHB-EGF. The cells were than treated with 1% formaldehyde at 40C for 10 min to cross-link proteins with DNA before harvesting. Cells were scraped off the plate, washed with ice-cold PBS, resuspended in 500 μl of hypotonic buffer (10 mM Tris-HCl, pH 7.4, 10 mM KCl, 1 mM DTT) and passed 20 times through a 25-gauge needle. Nuclei were spun down, resuspended in 200 μl SDS-containing lysis buffer (1% SDS, 10 mM EDTA, 50 mM Tris-HCl, pH 8.0 and protease inhibitors) and sonicated for two 30 s bursts separated by cooling on ice. After centrifugation, the supernatant was diluted 1:10 with immunoprecipitation buffer (50 mM Tris-HCl, pH 8.0, 150 mM NaCl, 5 mM EDTA, 0,5% NP40). Recombinant *Staphylococcus aureus* protein A was obtained as described previously [23]. Conjugation protocol of protein A to Sepharose CL-6B (Sigma-Aldrich, Missouri, USA) was modified from article [24]. The cell lysate was precleared by incubation with intact rabbit polyclonal serum for 1h at 40C and cleared by a further 1h incubation with protein A-Sepharose. The cleared lysates were incubated with anti-EGFR monoclonal antibody, anti-GST antibody, or intact rabbit polyclonal serum overnight. Immunoprecipitated complexes were collected by addition of protein A-agarose beads for 2h at 40C. Immunoprecipitates were washed once with RIPA buffer (150 mM NaCl, 50 mM Tris-HCl, pH 8.0, 0.1% SDS, 0.5% sodium deoxycholate, 1.0% NP40), once in high-salt wash (500 mM NaCl, 1.0% NP40, 0.1% SDS, 50 mM Tris-HCl, pH 8.0) and twice in TE buffer (10 mM Tris-HCl, pH 8.0, 1 mM EDTA). The beads were then treated with RNase (50 mg ml^-1^) for 30 min at 37°C. The cross-links were disrupted by heating at 65°C for 6 h and the DNA was then extracted with GeneJET DNA purification kit (Thermo Scientific, MA, USA). Specific sequences of cyclin D1 promoter in the immunoprecipitates were detected by PCR with primers Sence (5’-GAG GGG ACT AAT ATT TCC AGC AA-3’) and AntiSence (5’-TAA AGG GAT TTC AGC TTA GCA-3’).

Densitometry analysis was performed with Gels toolbox in FIJI software [22]. Prior to analysis background was subtracted with Subtract background tool in FIJI. Data plots was made with Excel 2007 (Microsoft Corporation, USA). Final pictures was collated with Adobe Illustrator (Adobe Corporation, USA).

## Results

### sHB-EGF induced EGFR nuclear importation

**Fig 1:**
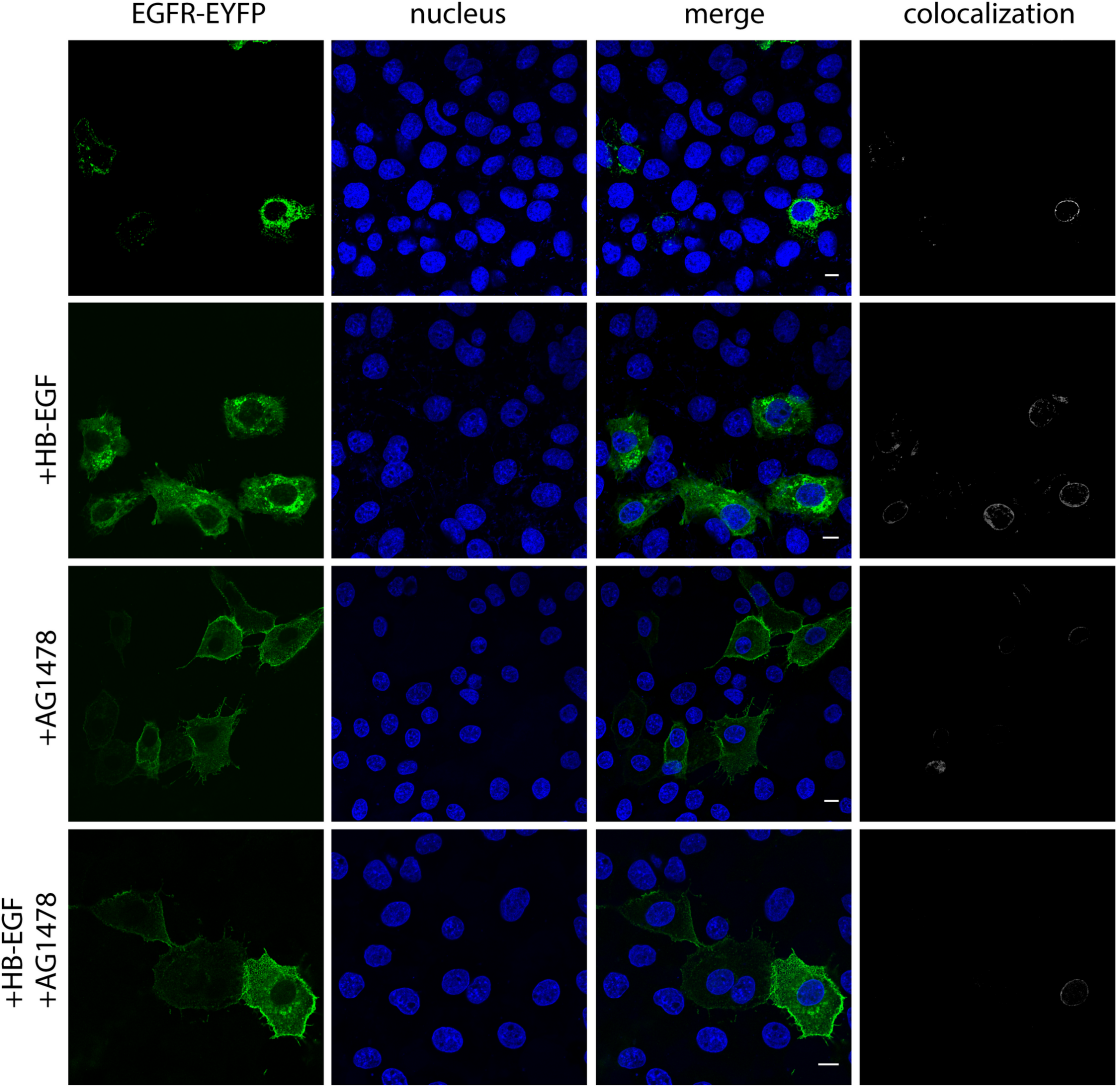
Subcellular localization of EGFR in sHB-EGF stimulated A431 cells. Immunofluorescent localization of EGFR in response to sHB-EGF treatment in concentration at 1,5 ug/ml during 30 min. (*Blue*, nucleus; *green*, EGFR-EGFP fusion protein). AG1478—EGFR tyrosine kinase inhibitor. Co-localization data analyzed with Fiji software. White scale bar corresponding to 10 um.

**Fig 2:**
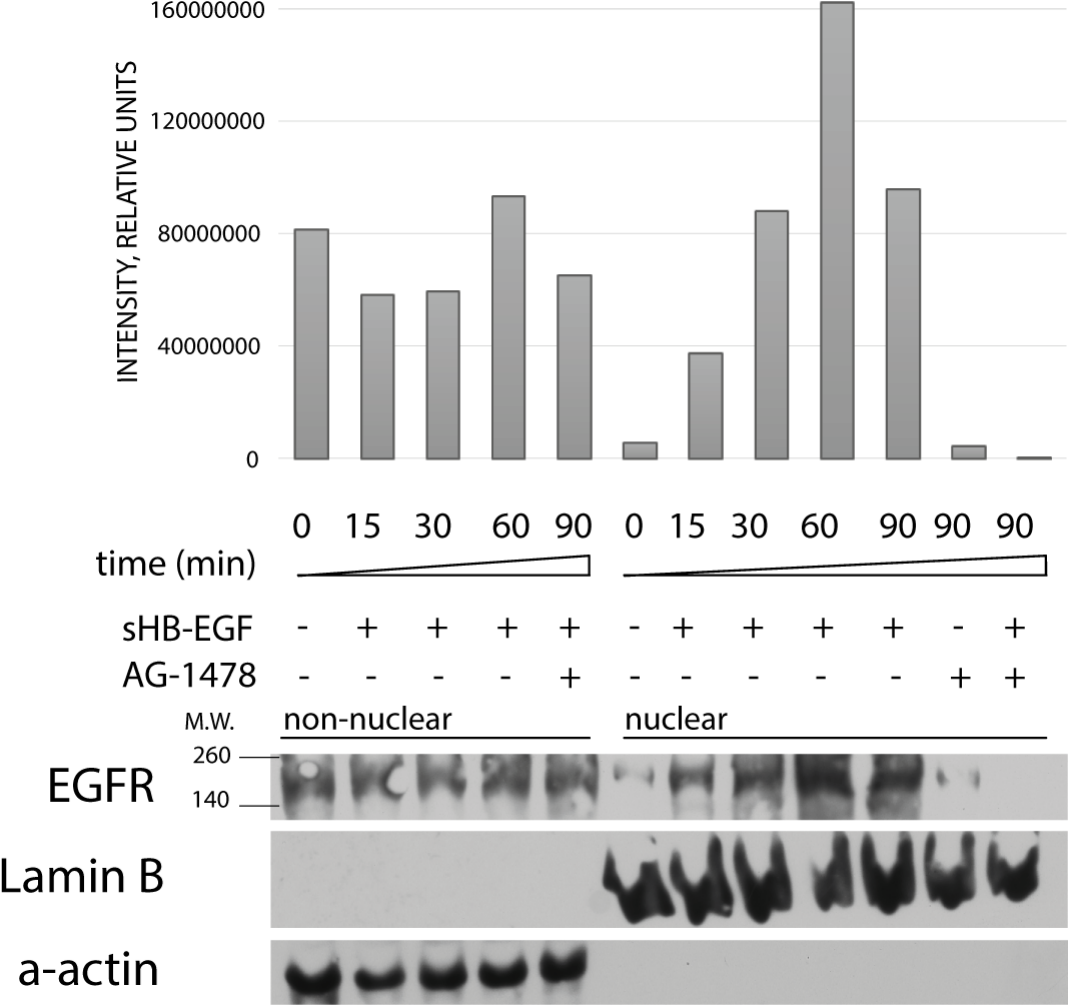
The EGFR localization in nuclear and cytoplasmic fractions after treatment of A431 cells with sHB-EGF. Non-nuclear and nuclear cellular extracts were subjected to western-blot analysis after sHB-EGF treatment in the absence or presence of EGFR tyrosine kinase inhibitor AG1478. Down-regulation of EGFR activity by kinase inhibitor AG1478 suppressed the EGFR nuclear localization in A431 cells under sHB-EGF treatment.

We used EGFR-overexpressing A431 cell line to study the appearance of nuclear EGFR in response to treatment with sHB-EGF. A431 cells were transfected with plasmid vector encoding EGFR labeled with enhanced yellow fluorescent protein (EGFR-EYFP). By means of confocal fluorescent microscopy, the EYFP-fused EGFR was found in the perinuclear region of A431 cells treated with sHB-EGF (Fig 1). Nuclear and cytoplasmic fractions from treated and untreated A431 cells were studied by Western-blot assay with anti-EGFR monoclonal antibody. The purity of the cytoplasmic fraction was controlled by enrichment for α-actin, whereas the purity of the nuclear fraction was controlled by enrichment for lamin B1. Both fractions showed no detectable cross-contamination. We found that nuclear EGFR level increased in a time-depended manner in response to treatment with sHB-EGF. Western-blot analysis showed that substantial amount of EGFR was accumulated in the nuclear fraction within 30–45 min after treatment of A431 cells with sHB-EGF. Addition of AG1478, the EGFR kinase inhibitor, prevented the nuclear translocation of EGFR under HB-EGF-stimulation (Fig 2). A small amount of the receptor was found in the nuclear fraction even without the added ligand, probably, due to autocrine stimulation.

### sHB-EGF and EGFR co-translocation to the nuclear envelope

**Fig 3:**
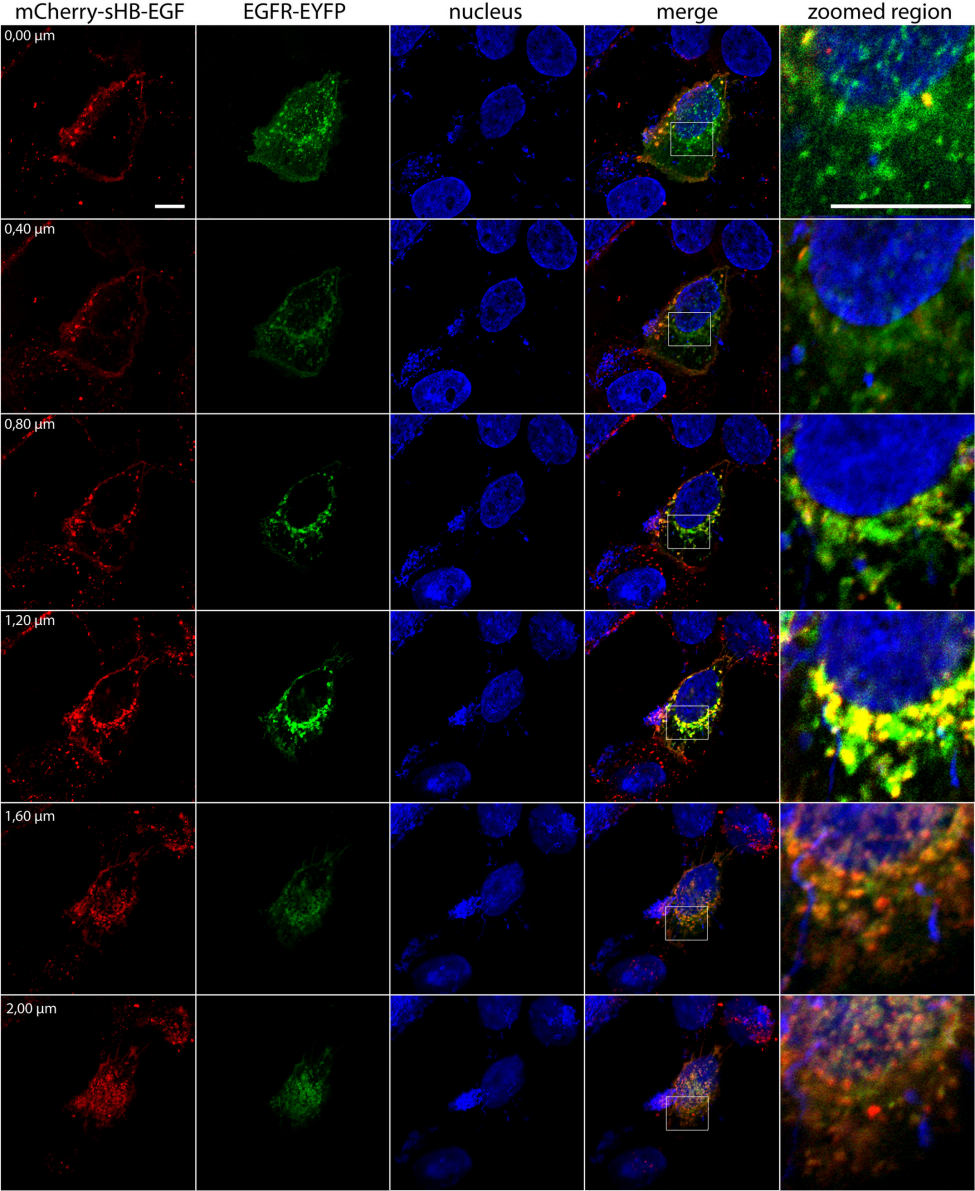
EGFR interaction with sHB-EGF and subcellular localization of their complex. Z-stack confocal image. A431 cells transfected with pEGFR-EYFP were stimulated with mCherry-sHB-EGF for 30 min. (mCherry-sHB-EGF *(Red)*; EGFR-EYFP *(green)*, sHB-EGF/EGFR ligand-receptor complex *(yellow)*). White scale bar corresponding to 10 um.

To investigate the ability of HB-EGF to form complexes with EGFR during its intracellular trafficking to the nuclear envelope we used fluorescent analogue of sHB-EGF labeled with mCherry (mCherry-sHB-EGF). A431 cells were transfected with plasmid pEGFR-EYFP and then were treated with mCherry-sHB-EGF. Hoechst 33342 was used to localize the nuclei. The ligand-receptor complex (yellow signal) was found in the perinuclear region as an overlap of green (EYFP) and red (mCherry) signals in Z-stack images (Fig 3). This data suggested that sHB-EGF interacts with EGFR during its translocation to the nuclear envelope.

### sHB-EGF is translocated to the nucleus by retrograde trafficking

**Fig 4:**
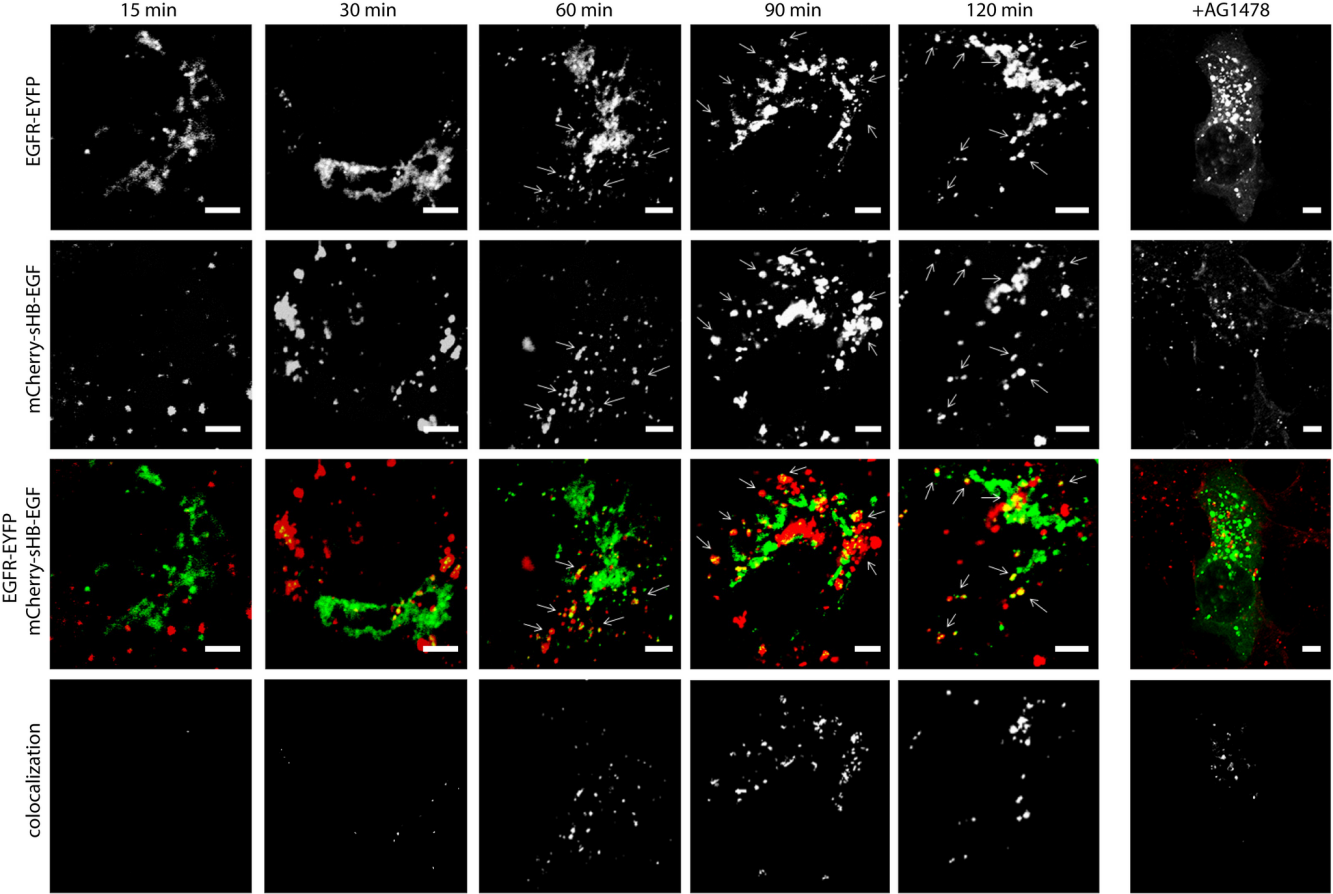
A time-dependent co-localization of mCherry-sHB-EGF with CGN-ER membranes in A431 cells. Cells were transfected with p23-EYFP and treated with sHB-EGF during different time periods. AG1478 was added during 60 min of sHB-EGF treatment. (mCherry-sHB-EGF *(Red)*, CGN-ER membranes *(green)*; co-localization of mCherry-sHB-EGF with CGN-ER membranes *(yellow* and *white)*). Co-localization data was calculated with FIJI software. White scale bar corresponding to 5 um.

We used the co-localization assay to investigate the intracellular mechanism of sHB-EGF-induced nuclear transportation of EGFR. A431 cells were transfected with the plasmid vector encoding an EYFP-fused major COPI-vesicle membrane protein p23. This protein is highly abundant in the cis-Golgi network (CGN) and constitutively circulates through the early secretory pathway [25]. Then the cells were treated with mCherry-sHB-EGF and studied for the presence of mCherry signal in CGN-ER membranes. Co-localization of mCherry-sHB-EGF with p23-EYFP suggested that ligand-receptor complex sHB-EGF/EGFR translocates to the nucleus via CGN-ER membranes (Fig 4). The strongest co-localization of mCherry and EYFP fluorescence signals has been observed in 60–90 min after treating the A431 cells with mCherry-sHB-EGF. This data indicated that sHB-EGF induced EGFR nuclear translocation run through CGN-ER membranes, probably, through COPI-coated vesicles pathway, also known as retrograde transportation pathway.

To investigate the role of EGFR kinase in the sHB-EGF- induced transportation of sHB-EGF/EGFR complex to CGN-ER membranes the cells were pretreated with EGFR kinase inhibitor AG1478. As shown in Fig 4, right panel, the translocation of mCherry-sHB-EGF to CGN-ER membranes was inhibited but not completely blocked with AG1478 after 60 min of treatment. This data suggested that COPI-mediated traffic of EGFR to the nucleus was, at least partly, EGFR kinase-independent.

### sHB-EGF is targeted to the nucleus as a part of EGFR/sHB-EGF ligand-receptor complex

**Fig 5:**
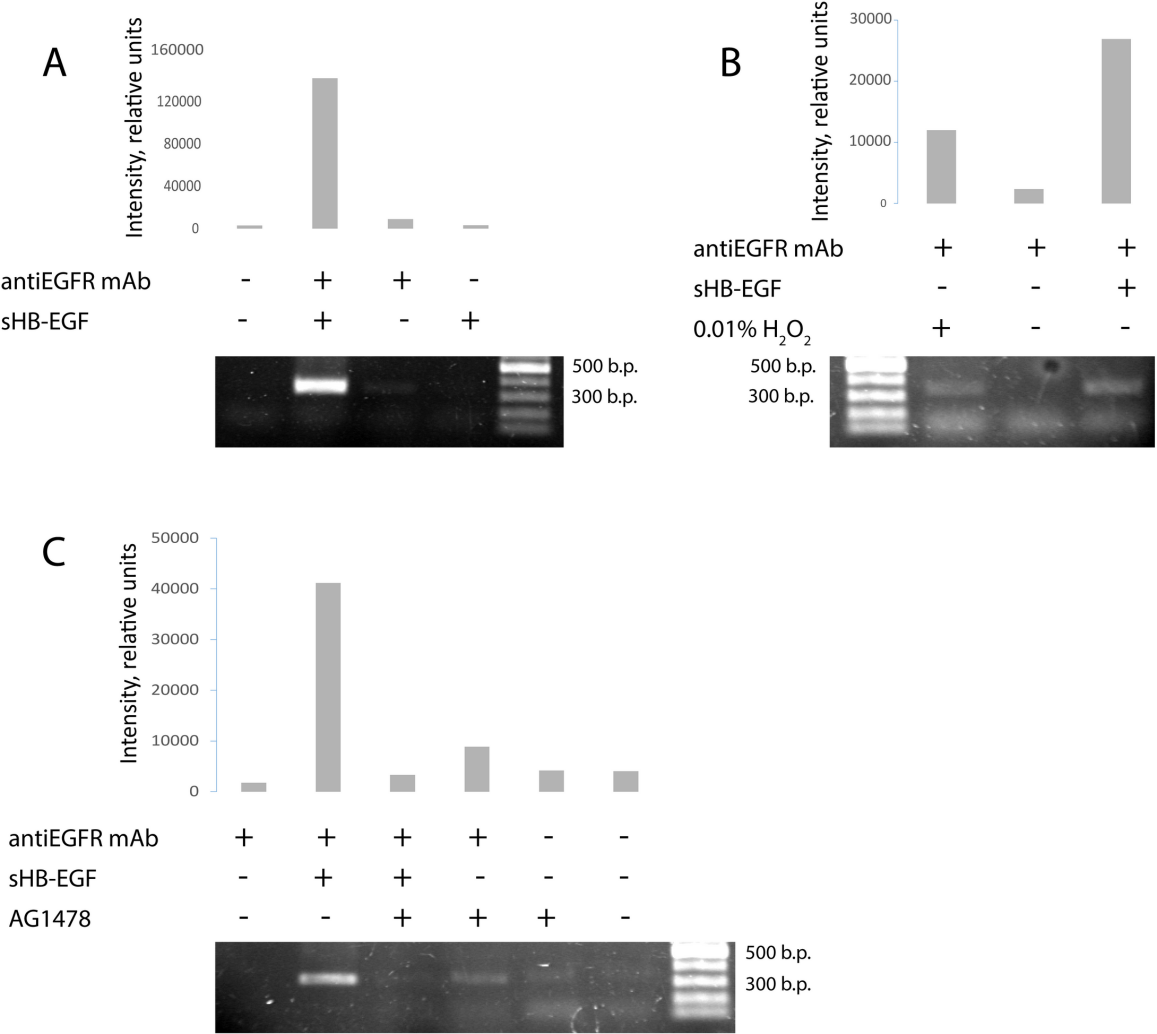
Chromatin immunoprecipitation with anti-EGFR monoclonal antibody. **A**, **B.** Nuclear localization of EGFR following sHB-EGF (A) and H_2_O_2_ (B) treatment. Increased association of nuclear EGFR and the cyclin D1 promoter region following sHB-EGF treatment is shown. **C**, Down-regulation of EGFR activity by kinase inhibitor AG1478 suppressed the association of EGFR with the cyclin D1 promoter in A431 cells under sHB-EGF treatment.

The promoter region of cyclin D1 gene is considered as one of the most important EGFR nuclear targets [10]. We used ChIP assay to investigate if sHB-EGF stimulates EGFR interaction with cyclin D1 promoter. For this purpose, the nuclei of sHB-EGF- treated cells were sonicated and, after precipitation of disrupted chromatin with specific anti-EGFR monoclonal antibody, the cyclin D1 promoter region was amplified by PCR. Non-immune rabbit polyclonal serum was used for a negative control. As shown in Fig 5A, the nuclear EGFR was found associated with the endogenous cyclin D1 promoter after stimulation of A431 cells with sHB-EGF (500 ng ml-1).

Typically, the level of nuclear EGFR increases in response to the cell treatment with ROS, heat, radiation or cisplatinum [26]. Therefore, we used A431 cells treated with 0.01% H2O2 as a positive control for the translocation of EGFR to the nucleus. As shown in Fig 5B, sHB-EGF induced EGFR nuclear import similarly to H2O2. The EGFR kinase inhibitor AG1478 prevented EGFR interaction with cyclin D1 promoter upon sHB-EGF treatment (Fig 5C), but did not inhibit it completely. This observation was in accordance with the data presented in Fig 4 and indicated that ligand-driven association of EGFR with cyclin D1 promoter region was, at least in part, kinase-independent.

**Fig 6:**
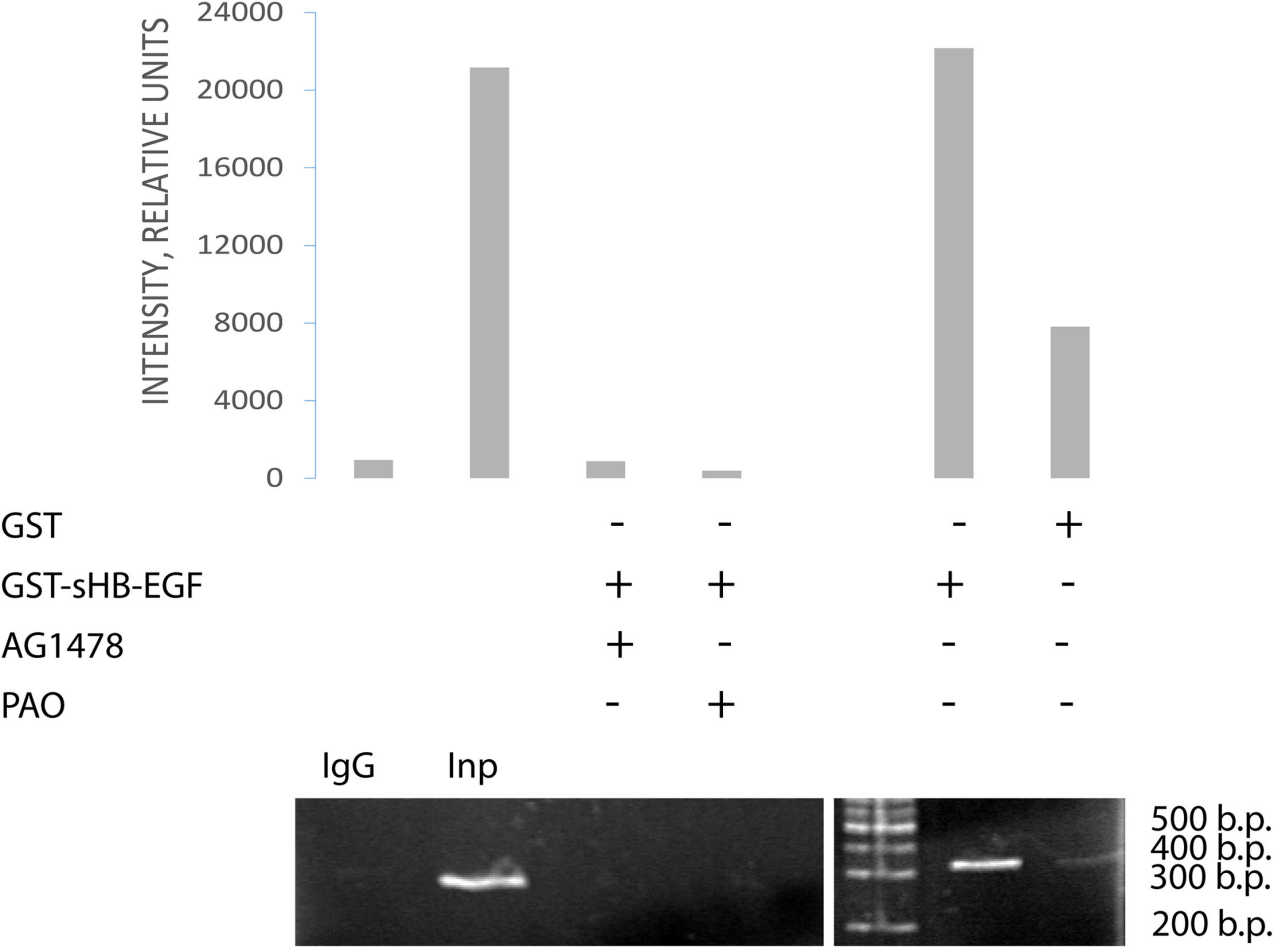
Chromatin immunoprecipitation with anti-GST antibodies to identify the nuclear localization of GST-sHB-EGF. Down-regulation of EGFR activity by kinase inhibitor AG1478 and endocytosis inhibitor (PAO) suppressed the association of GST—sHB-EGF with the cyclin D1 promoter in A431 cells. *Inp*—input nuclear DNA.

The above-mentioned data suggested that sHB-EGF was transported to the nucleus in association with EGFR and could be detected there as a part of the ligand-receptor complex. We performed ChIP assay based on ligand-receptor interactions to detect sHB-EGF associated with EGFR in the nucleus. A431 cells were treated with glutatione S-transferase-fused sHB-EGF (GST-sHB-EGF) or with GST alone (negative control). The inhibitors of the EGFR-kinase (AG1478) or of endocytosis (phenylarsine oxide, PAO) were used to block internalization of surface EGFR. ChIP assay performed with the nuclear lysates of A431 cells using GST-sHB-EGF-specific antibody revealed that sHB-EGF remained bound to EGFR when EGFR associated with cyclin D1 promoter. Precipitation of EGFR/GST-sHB-EGF complex with anti-GST antibody resulted in amplification of cyclin D1 promoter region in GST-sHB-EGF-treated A431 cells butwhen the cells were treated only with GST or with GST-sHB-EGF in the presence of AG1478 or PAO the promoter region was not revealed (Fig 6).

## Discussion

The biological activity of HB-EGF is usually attributed to its soluble form, which acts by binding to the EGFR or ErbB4 on the cell surface. ProHB-EGF has already been reported to localize within the nucleus, but authors described its nuclear localization independently on binding to the receptor [15]. The present study shows for the first time that nuclear translocation of EGFR can be induced by the soluble form of HB-EGF in human cervical cancer A431 cells. Using western-blot analysis we showed that ligand-dependent nuclear translocation of EGFR occurred after 30–60 min upon treatment of cells with sHB-EGF. Application of EGFR kinase inhibitor AG-1478 prevented EGFR appearance in the nuclear fraction (Fig 2). However, a small amount of EGFR was detected in the nuclei of untreated A431 cells, possibly, due to autocrine EGFR stimulation [27].

Obtained data, presented on Fig 5 indicated that sHB-EGF-stimulated nuclear EGFR could interact with cyclin D1 promoter region in the nucleus. We showed that nuclear EGFR was associated with cyclin D1 promoter region in sHB-EGF-treated cells (Fig 5A) and this association was prevented by application of EGFR kinase inhibitor AG-1478 (Fig 5C). The absence of PCR signal in unstimulated cells led us to the conclusion that small amount of nuclear EGFR detected by western-blot in unstimulated cells (Fig 2) was not able to interact with cyclin D1 promoter region. On the other hand, the visible PCR band appeared after AG-1478 treatment of non-stimulated cells confirmed the previous findings that EGFR can be transported to the nucleus in response to inhibition of tyrosine kinase-dependent signaling pathway [28].

Numerous EGFR ligands can be translocated into the nucleus in association with the receptor. For example, EGF/EGFR complex was detected in the nucleus in MDA-MB-468 cells [10]. We showed that sHB-EGF was transported to the nuclear envelope as a part of sHB-EGF/EGFR ligand-receptor complex to associate with cyclin D1 promoter in a tyrosine phosphorylation-dependnt manner. The biological role of receptor-associated ligand in the nucleus remains to be investigated; we hypothesize that sHB-EGF could maintain appropriate conformation of EGFR in the ligand-receptor complex, which serves as a transcriptional regulator.

It is difficult to imagine that an entire membrane-embedded EGFR escapes from the lipid bilayer to enter the nucleus; however, recent studies provide a comprehensive model of transmembrane EGFR transport to the nucleus via retrograde trafficking [29]. We showed that sHB-EGF-driven EGFR passes through CGN-ER membranes on the way from the cell surface to the nucleus and, therefore, remains in the membrane-embedded form throughout the trafficking pathway (Fig 4). Moreover, predominant co-localization of sHB-EGF/EGFR complex with nuclear membranes supports the idea that EGFR realizes its intranuclear activity in membrane-embedded form at the nuclear periphery (Figs 1 and 3). Since inhibition of EGFR kinase only partially reduced co-localization of sHB-EGF- EGFR complex with CGN-ER membranes, we suggest that certain EGFR kinase-independent mechanisms are involved in sHB-EGF-stimulated retrograde EGFR traffic. However, further nuclear association of sHB-EGF/EGFR complex with cyclin D1 promoter was completely inhibited with AG-1478; therefore, tyrosine kinases seemed to play a critical (crucial) role in this association.

EGFR family members play a prominent role in signal transduction. However, EGFR kinase also has some non-canonical functions, such as transcriptional regulation, DNA synthesis and DNA repair in the nucleus [30]. It is known that nuclear translocation of EGFR family ligands promotes cell survival and, therefore, is associated with enhanced resistance to radiation, chemotherapy and anti-EGFR therapies [31]. Ability of soluble ligands to increase the levels of nuclear EGFR in cancer cells can explain the acquired resistance to anticancer drugs. Further studies aimed to reduce the level of nuclear EGFR may be important for conferring resistance to anti-EGFR kinase target agents, chemotherapy, ionizing radiation etc.
